# Synthetic Polymers for Organ 3D Printing

**DOI:** 10.3390/polym12081765

**Published:** 2020-08-07

**Authors:** Fan Liu, Xiaohong Wang

**Affiliations:** 1 Center of 3D Printing & Organ Manufacturing, School of Fundamental Sciences, China Medical University (CMU), No. 77 Puhe Road, Shenyang North New Area, Shenyang 110122, China; liufan-sky@163.com; 2 Department of Orthodontics, School of Stomatology, China Medical University, No. 117 North Nanjing Street, Shenyang 110003, China; 3 Center of Organ Manufacturing, Department of Mechanical Engineering, Tsinghua University, Beijing 100084, China

**Keywords:** three-dimensional (3D) printing, synthetic polymers, rapid prototyping (RP), organ manufacturing, implantable bioartificial organs, regenerative medicine

## Abstract

Three-dimensional (3D) printing, known as the most promising approach for bioartificial organ manufacturing, has provided unprecedented versatility in delivering multi-functional cells along with other biomaterials with precise control of their locations in space. The constantly emerging 3D printing technologies are the integration results of biomaterials with other related techniques in biology, chemistry, physics, mechanics and medicine. Synthetic polymers have played a key role in supporting cellular and biomolecular (or bioactive agent) activities before, during and after the 3D printing processes. In particular, biodegradable synthetic polymers are preferable candidates for bioartificial organ manufacturing with excellent mechanical properties, tunable chemical structures, non-toxic degradation products and controllable degradation rates. In this review, we aim to cover the recent progress of synthetic polymers in organ 3D printing fields. It is structured as introducing the main approaches of 3D printing technologies, the important properties of 3D printable synthetic polymers, the successful models of bioartificial organ printing and the perspectives of synthetic polymers in vascularized and innervated organ 3D printing areas.

## 1. Introduction

The number of people in the world suffering from organ dysfunction, defect or other deformities is rapidly increasing because of the aging population, chronic and acute diseases and traffic accidents [1]. At present, autologous transplantation is the best way to repair the intrinsic defects, damaged parts and organ failure. However, it is severely limited by the shortage of homogeneous transplants from the patients themselves. Orthotopic (or allogenic) organ transplantation is an effective approach to curing the deformities. Nevertheless, it is strictly overrated by the vast deficiency of allografts, life-long side effects of immunosuppressive drugs, and extremely high cost of surgical operations [2,3]. 

Three-dimensional (3D) printing technologies, previously named as additive manufacturing (AM), solid freeform fabrication (SFF), and rapid prototyping (RP), are enabling manufacturing processes that automatically produce complex structures directly from computer-aided design (CAD) models with high resolution and sophistication. These technologies are based on a layered manufacturing paradigm that builds solid objects by incremental material deposition and fusion of thin cross-sectional layers. By breaking down complex 3D shapes into simpler two-dimensional (2D) layers, the assembling of very complex structures can be dramatically simplified under the instructions of CAD models. At present, 3D printing technologies are considered as the most convenient and reliable techniques for manufacturing bioartificial organs with multiple types of cells as well as other biomaterials [4,5]. 

Over the past decade, 3D printing technologies have developed quickly and have been applied to almost every biomedical field. Compared with traditional manufacturing technologies, these technologies have many advantages in rapid, precise and custom-built biomedical devices, such as large scale-up scaffolds, living tissues and bioartificial organs. A growing number of living tissues and organs, with a wide range of biomaterials, go-through channels and vascular/neural networks, have been constructed utilizing cell-laden polymeric hydrogels or solutions [6,7,8]. 

Polymers as the main components of ‘bioinks’ have played a critical role in organ 3D printing during the layered 3D construction processes [9]. Most of the ‘bioinks’ are cell-laden polymeric hydrogels which are usually formed through physical (reversible), chemical (reversible or irreversible) or biochemical (irreversible) crosslinking of homopolymer or copolymer solutions. Cell behaviors within the polymeric hydrogels can be controlled through changing the physical and chemical properties of the employed polymers. The polymeric hydrogels for organ 3D printing include natural and synthetic polymers and their combinations. Natural polymeric chains are full of bioactive groups, which can provide a benign and stable environment for cells, especially stem cells, to grow, migrate, proliferate, and/or differentiate inside [10,11]. Synthetic polymeric networks are comprised of repeatable inert units. They are usually superior to natural polymers in terms of mechanical properties and immunogenic responses. 

In particular, several series of unique automatic and semi-automatic bioartificial organ manufacturing technologies have been exploited in our own group with the proper integration of 3D printing technologies with biomaterials, especially polymers. With these unique technologies we have solved all the bottleneck problems, such as large scale-up tissue/organ manufacturing [12,13,14], long-term preservation of bioartificial tissues/organs [15,16], step-by-step adipose-derived stem cell (ASC) differentiation in 3D constructs [17,18], hierarchical vascular/nerve network construction with a fully endothelialized inner surface and anti-suture capability [19,20,21,22], in vitro metabolism model establishment [23,24], high-throughput drug screening [25,26,27], biocompatibilities of implanted biomaterials [28,29,30,31], which have troubled tissue engineers, biomaterial researchers, pharmaceutists and other scientists for more than seven or more decades. Several polymers have played essential and ubiquitous roles in bioartificial organ manufacturing with the incorporation of multiple cell types, stem cells/growth factors, hierarchical vascular and neural networks with anti-suture and anti-stress functions.

We have previously reported natural polymers for organ 3D printing [32]. In this review, some commonly used synthetic polymers with excellent biocompatibility, 3D printability, biodegradability and mechanical properties for organ 3D printing technologies are presented. In particular, the intrinsic/extrinsic properties of the synthetic polymers for bioartificial organ 3D printing are outlined. Typical 3D printing models for vascularized and neuralized (or innervated) organ manufacturing are highlighted.

## 2. Synthetic Polymers for 3D Printing

**Figure 1:**
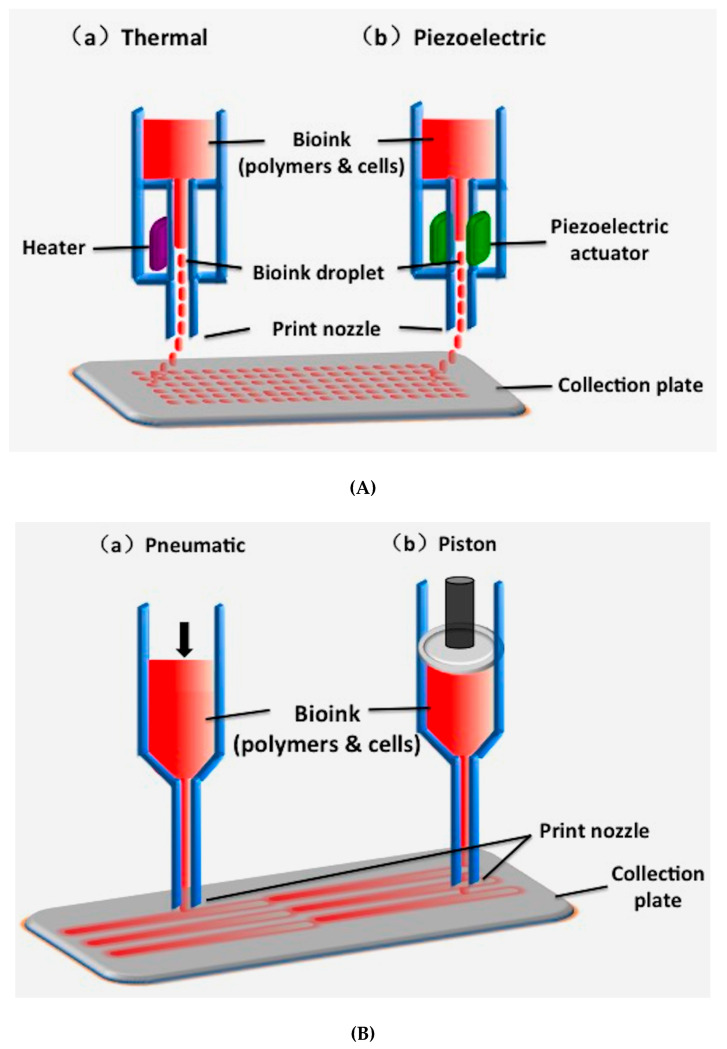

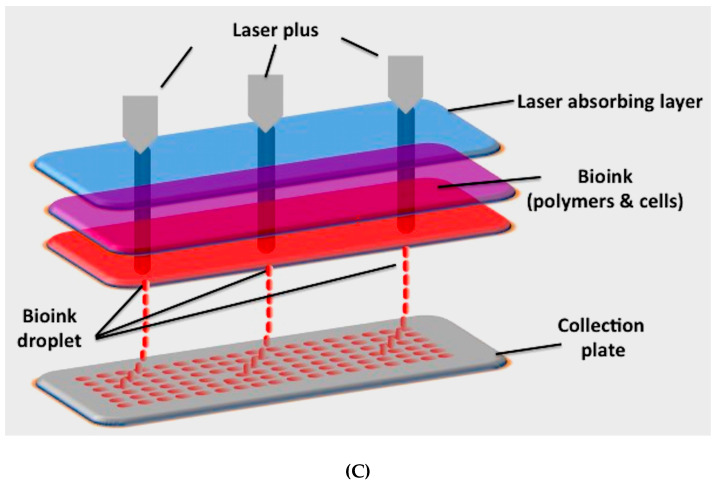
Schematic diagrams of three major types of three-dimensional (3D) printing technologies: (**A**) inkjet-based bioprinting (a: heater; b: piezoelectric actuator); (**B**) extrusion-based printing (a: pneumatic; b: piston); (**C**) laser-assisted bioprinting [33].

Given that working principles, there are three major types of 3D printing technologies that use synthetic polymers as the processing (or starting) materials: inkjet-based 3D printing, extrusion-based 3D printing and laser/light-assisted 3D printing (Figure 1) [33]. Among these technologies, fused deposition modeling (FDM), extrusion-based printing (EBB), stereolithography (SLA) and digital light processing (DLP) are the most commonly used technologies for porous scaffold manufacturing. Meanwhile, the most commonly printed synthetic polymers include poly (lactic acid) (PLA), poly (glycolic acid) (PGA), polylactic-co-glycolic acid (PLGA), polyurethane (PU), and polycaprolactone (PCL) [34,35,36,37,38,39,40]. The most important advantage of these technologies in organ 3D printing is that they facilitate the control of porosity and interconnectivity of the micro/macropores or go-through channels in the polymeric constructs. In the following section, several frequently used 3D printing technologies employing synthetic polymers as the processing (or starting) materials are introduced. 

### 2.1. Inkjet-Based 3D Printing

Inkjet-based 3D printing is a non-contact AM technique adapted from industrial 2D printers, in which droplets of building materials are selectively deposited. Example materials include photopolymers and waxes. By changing the content of ‘inks’, cells and polymers can also be patterned into desired shapes (Figure 1A).

The drop-on demand inkjet printers are the most common ones, which consist of thermal, piezoelectric, and electrostatic inkjet nozzles [41]. Inkjet printers are normally used for printing tissue engineering scaffolds for cell seeding. Recently, different inkjet printheads with multiple nozzles have been developed to increase the printing speed and construct size [42].

Despite holding some merits for biomaterial printing, there are many limitations of inkjet-based printing technologies to be used for organ manufacturing. These limitations include low polymer viscosity (ideally below 10 centipoise), low cell density (less than 10 million cells/mL), and low structural heights (less than 10 million cells/mL) [43]. To provide a higher polymer concentration or cell density, crosslinking agents are often used, resulting in some drawbacks, such as reduced printing processes and changed material properties [44]. Over the last decade, significant studies have been conducted for in vitro and in situ printing living tissues, including cartilage and skin, and moderate progress has been achieved compared with extrusion-based 3D printing technologies (Figure 1B) [45].

### 2.2. Fused Deposition Modeling (FDM)

**Figure 2:**
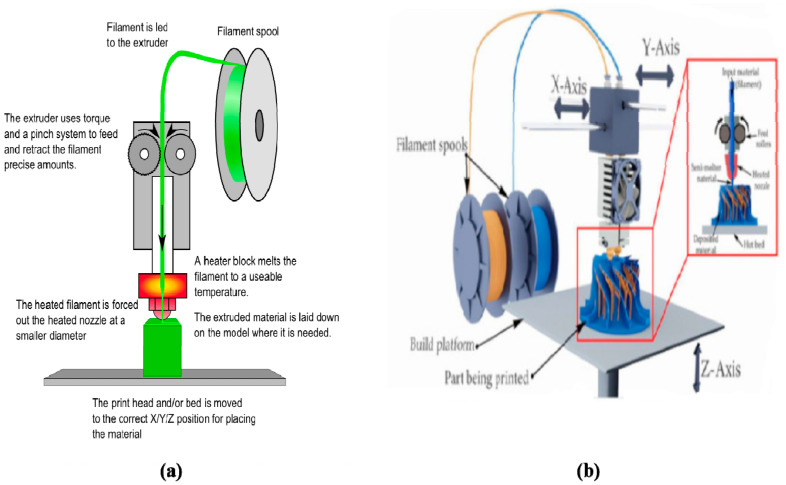
Schematic illustration of fused deposition modeling (FDM) processes using synthetic polymers, such as acrylonitrile-butadiene-styrene copolymer (ABS), polyamide, polycarbonate, polyethylene and polypropylene: (**a**) one nozzle three-dimensional (3D) printing; (**b**) two nozzle 3D printing [46,47].

FDM is the layered deposition of molten thermoplastic polymers (i.e., thermoplastics) through one or more heated extrusion heads with a small orifice in a specific lay-down pattern (Figure 2) [46,47]. It is a kind of specific extrusion-based 3D printing technology, known by the technical term ‘thermoplastic extrusion’. In FDM, one of the traditional methods is to melt thermoplastic polymers into semi-liquid states and extrude the semi-liquid polymers onto the platform in layers. The polymers can be supplied in filament or pellet forms. When the thermoplastic polymers are heated to a temperature above their melting points, they become fluidic and flow from the nozzles. The plastic polymers harden immediately after flowing from the nozzles and bound to the layer below. Once a layer is built, the platform lowers, and the extrusion nozzle deposits another layer. The printed layers fuse together in layers. The layer thickness and vertical dimensional accuracy is determined by the nozzle diameter, which ranges from 0.033 to 0.127 cm. In the X–Y plane, a 0.003 cm resolution can be achieved.

A range of synthetic polymers, such as acrylonitrile-butadiene-styrene copolymer (ABS), polyamide, polycarbonate, polyethylene (PE) and polypropylene, have been printed by FDM. Some of the polymers (e.g., ABS) are unbiodegradable and the 3D-printed products are for industrial uses, such as toys, clothes and guns. There is no theoretical restriction on the compositional gradients in all three dimensions for FDM technologies since multiple extrusion nozzles can be easily equipped, each with a different polymer. In the early 2000s, FDM technologies were the most popular industrial AM technologies worldwide [46,47].

The most important criteria for FDM technology evaluation are heat transfer characteristics and rheological behaviors of the selected polymers. Thermoplastics, such as polyvinyl chloride (PVC), polyacrylamide, PCL, PE, PLGA are the commonly used synthetic polymers for soft matter printing [48,49]. For example, PCL has been normally used for tissue engineering scaffold preparation due to its low melting temperature (i.e., glass transition temperature) of 60 °C, and high thermal stability [50]. 3D printing of PLGA, with a glass transition temperature of 45–50 °C and melting temperature of >120 °C, has proven to be challenging at a higher extrusion temperature [51,52]. One of the captivating advantages of FDM technologies in AM fields is the creation of complex scaffolds with good mechanical strength and geometric accuracy, while one of the fatal shortcomings of FDM technologies in organ 3D printing areas is that cells, growth factors, and other bioactive agents cannot be printed directly under high melting temperatures. 

Additionally, the printing nozzle temperature of FDM can be as high as 500 ℃ for some special engineering plastics, such as polyetheretherketone (PEEK), poly(oxy-1,4-phenylenecarbonyl-1,4-phenylenecarbonyl-1,4-phenylene) (PEKK), polyphenylene sulfone resins (PPSU), polyetherimide (PEI) and PE. Correspondingly, the printing resolution can be as high as 1 µm. Nevertheless, most of these synthetic polymers are poisonous to the human body. They are hardly fit to be used as building materials for organ 3D printing containing living cells and bioactive agents.

### 2.3. Extrusion-Based Printing 

Similarly to FDM, extrusion-based printing is an automatic fluid dispensing system, in which polymeric materials are selectively dispensed through one or more nozzles or orifices. Different from FDM, the extrusion-based extrusion processes do not involve any heating procedures unless especially necessary. Polymer solutions or hydrogels with or without cells, growth factors, and other bioactive agents, can all be extruded through nozzles by pneumatic pressure or physical force (i.e., a piston or screw) in a controllable manner [53]. The printing system generates continuous filaments under the control of CAD models. With the development of this of kind 3D printing technologies, it is possible to deposit living cells along with biocompatible polymers with very high cell densities. The solidification of polymer solutions or hydrosols is achieved through a series of physical and chemical procedures, such as sol-gel transformation (i.e., physical crosslinking), polymerization, chemical crosslinking, and enzymatic reaction [4,5,6]. 

Over the last decade, extrusion-based printing has been the most developed technology for biomedical applications. Especially, multi-nozzle extrusion-based printing and four-dimensional (4D) printing technologies have evolved quickly for complex object production. For example, a N-isopropylacrylamide-based stimuli-responsive pre-gel solution (NIPAM-based ink) and an acrylamide-based non-responsive pre-gel solution (AAM-based ink) in a supporting viscous liquid (carboxymethyl cellulose solution) are printed by polymerizing the printed structures using ultraviolet (UV) light irradiation [54,55]. It was found that the positions of the multiple polymers changed sophistically according to the physical properties of the ‘inks’ (i.e., polymer solutions or hydrogels) and nozzle states (e.g., diameter, position, and speed). For most of the polymeric hydrogels, the width of the printed filaments is mainly influenced by the fluid flow rate and nozzle moving speed [12,13,14,15,16,17,18]. 

The advantages of extrusion-based printing in organ 3D printing include high cell densities, large scale-up structures and extremely sophisticated compositions. A large number of biomaterials, including cells, growth factors, and other bioactive agents, can be simultaneously deposited with polymeric solutions or hydrogels. With the increase of extrusion nozzles, a variety of heterogeneous constructs with multiple polymer types and cell types can be constructed. Many researchers have addressed the effects of extrusion process parameters, such as speed of 3D dispensing, pressure, temperature, nozzle size, viscosity and shear thinning of polymeric solutions or hydrogels, on cell viabilities [56,57,58,59,60]. 

In our group, we have created a series of extrusion-based 3D printing technologies for complex bioartificial organ manufacturing. A great deal of pioneer work has been performed in our laboratory using our homemade 3D printers. In particular, multiple cell types in some gelatin-based hydrogels have been assembled at temperatures of 1–10 °C to obtain living tissues/organs with desired geometrical structures, biochemical components and physiological functions. The living tissues/organs have had a far-reaching impact on other pertinent disciplines, such as biomaterials (e.g., biomaterial integration/implantation), cell transplantation, high throughput drug screening, tissue engineering and regenerative medicine, energy metabolism model establishment, and pathological mechanism analyses [61,62,63]. 

A critical limitation of the 3D-printed living cells/natural polymers for bioartificial organ manufacturing are the notorious weak mechanical properties of the products without anti-suture and anti-stress functions. A practicable solution is to integrate synthetic polymers into the available constructs. However, most synthetic polymers do not have sol-gel transition (or phase transformation, traditionally glass transition) temperatures between 1–10 °C. To overcome this shortcoming, we have explored several series of low-temperature deposition manufacturing technologies equipped with one, two or more extrusion nozzles. Under low-temperature conditions, such as minus 20–30 °C, both natural and synthetic polymers are frozen and distributed into predefined space to form elaborated 3D constructs. In particular, both organic and inorganic solvents in the natural and synthetic polymer solutions or hydrogels can be easily substituted through a cell culture medium after 3D printing. Otherwise, organic solvents in the synthetic polymer systems can be evaporated through phase separation (i.e., freeze-drying) techniques. 

### 2.4. Stereolithography (SLA)

SLA is a solid freeform, nozzle-free, laser- or light-assisted 3D printing technology based on photosensitive macromolecule (i.e., polymer) formulation [64]. It is a multilayer procedure through the selective photoinitiated curing reaction of a low-molecular-weight pre-polymer, additives and photoinitiators. The instrument setup consists of a vessel that contains a photosensitive resin, a moveable platform on which the model is built, and a computer-controlled laser beam that is operated in a defined CAD pattern. Optimal digital micromirror devices can work within wavelengths between 385–405 nm with an expected lifetime of 2000 h when exposed to a radiation of 10 w/cm^2^ light intensity [65]. Compared with other 3D printing technologies, SLA can produce complex structures with high resolution and accuracy. 

**Figure 3:**
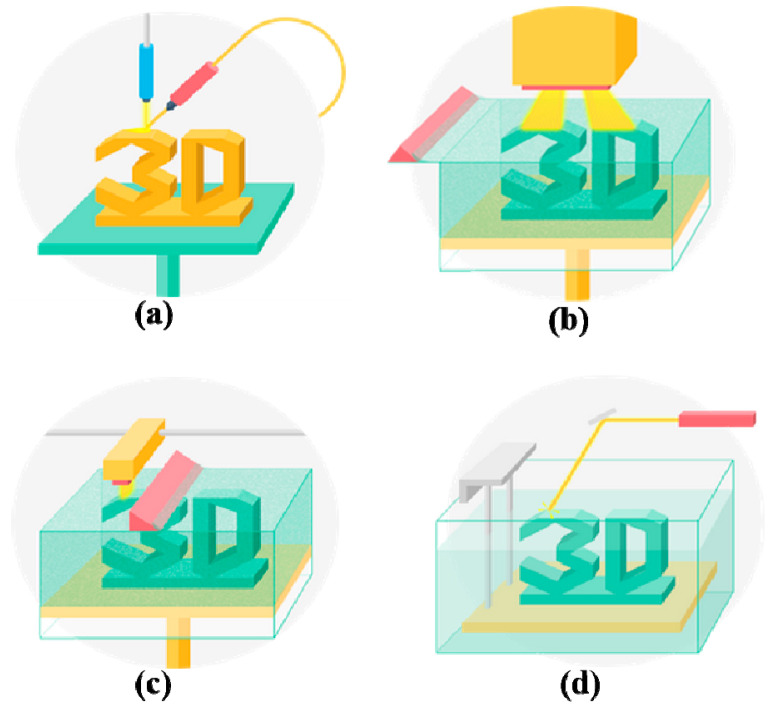
Schematic depiction of various stereolithography (SLA) technologies: (**a**) laser sintering; (**b**) two-photonpolymerization (TPP); (**c**) powder bed fusion; (**d**) vat photopolymerization.

There are many types of SLA, in which focused thermal energy can be used to fuse materials by melting as they are being deposited. Among these types, ’focused thermal energy’ means that an energy source (e.g., laser, electron beam, heat, or plasma arc) is focused to melt the materials being deposited. If the printed materials are in powder state, it is also called ‘powder bed fusion’, in which thermal energy selectively fuses the regions of a powder bed. If a liquid photopolymer in a vat is selectively cured by light-activated polymerization, this process is called vat photopolymerization. These types of SLA all belong to direct energy deposition manufacturing (Figure 3). 

The use of laser-assisted printing technology for 2D biomaterial printing can be traced back to 2003 [66], while the development of 3D structures was reported in 2011 [67]. The ability of 3D-printed cells to form real tissues was demonstrated in 2012 [68]. Later experiments showed that while higher laser energy leads to increased cell fatality, increasing polymer viscosity results in increased cell viability [69]. 

It is noticed that one important requirement of SLA technologies is that the processing materials must have photocurable moieties. Thus, functionalized oligomers, star/branched polymers, hyperbranched polymers are common building blocks for achieving desired geometries [70]. Light-sensitive hydrogels, such as polyethylene glycol diacrylate (PEGDA) and methacrylated gelatin (GelMA), can also be printed using the devices in a layer-by-layer manner. The layer thickness depends on the printer model standards, which could range from 15 to 150 μm. Cell viability could reach 90% within a short printing time, approximately 30 min [65]. The disadvantages of SLA technologies in organ 3D printing are the high cost of the devices and the cytotoxicity of the lights and photoinitiators [71]. Compared with extrusion-based 3D printing, SLA is a time-consuming, high cost and ineffective method for bioartificial organ manufacturing. 

### 2.5. Digital Light Processing (DLP) 

DLP is a light-assisted printing technology, that is similar to SLA. Both the DLP and SLA technologies utilize light to selectively crosslink photopolymers in a layer-by-layer pattern to build freeform objects. The light source is one of the main differences between SLA and DLP. Generally, DLP uses a digital mirror device chip, which is composed of approximately one million micromirrors, to regulate the UV light or visible light [72], while image creation through SLA usually uses an arc lamp. 

Similarly to DLP, another AM process in which a liquid bonding agent is selectively deposited to join powder materials is called binder jetting. In this process, light is substituted by a liquid binder [73,74]. Meanwhile, the powder materials can be various synthetic polymers. 

In DLP, each mirror represents one or more pixels in the projected image. The number of mirrors corresponds to the resolution of the projected images. The resolution of DLP is determined by the focal size of the light beam from each micromirror at micron scale (10–50 µm) [75]. The whole layer of the fabricated construct is produced in one exposure step, which is different from the point-by-point pattern of SLA. Thus, the build time is considerably shorter than that of SLA. Due to the short printing time and nozzle-free printing technique, cell viability could reach more than 90% [76]. Compared with SLA, DLP is less affected by oxygen inhibition, because the object is pulled up from the liquid resin, rather than down and further into the liquid photo-polymeric system and is not in direct contact with air. Furthermore, both SLA and DLP need support structures to manufacture objects with large volume. The support structures must be mechanically removed from the finished parts after 3D printing [77,78]. 

### 2.6. Aerosol Jet 3D Printing

**Figure 4:**
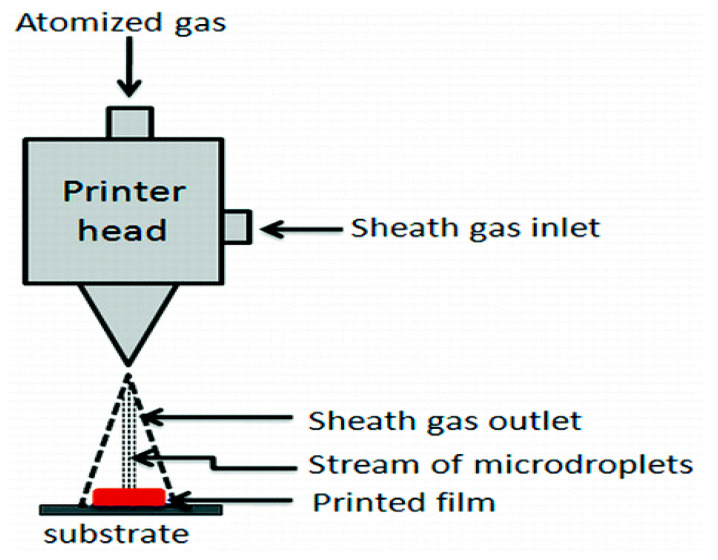
Working principle of aerosol jet three-dimensional printing [79].

**Figure 5:**
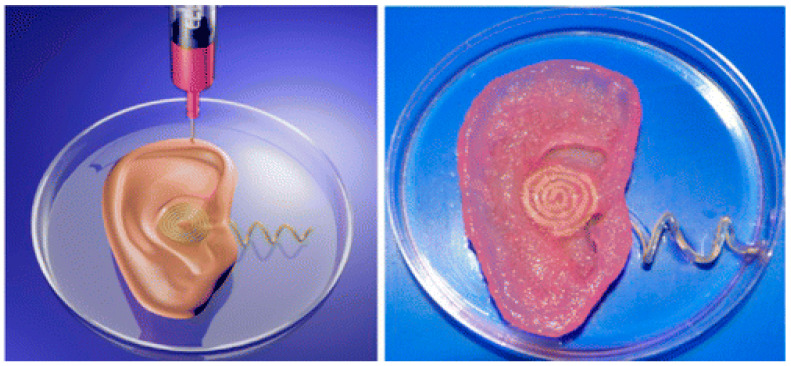
Three-dimensional printed bionic ears [80].

Aerosol jet 3D printing is a new non-contact direct-writing technique, similar to inkjet-based 3D printing (Figure 4) [79]. It has recently emerged as a promising technique for the manufacturing of electrochemical energy conversion and storage devices, such as batteries, fuel cells, and supercapacitors. In aerosol jet 3D printing, it is required to control a number of process parameters, including the flow rate of the carrier gas that transports the aerosol mist to the platform (or substrate), the flow rate of the sheath gas that collimates the aerosol into a narrow beam, and the speed of the stage that transports the substrate beneath the beam. Most of the currently available printing materials are metals. A few synthetic polymers, such as poly(3-hexylthiophene), poly(N-vinylcarbazole) and poly[N-9-heptadecanyl-2,7-carbazole-alt-3,6-bis(thiophen-5-yl)-2,5-diethylhexyl-2,5-dihydropyrrolo-[3,4–]pyrrole-1,4-dione], have been printed into solar cells. Few examples of aerosol jet 3D printing technologies have been explored for biomedical applications. Recently, interest in flexible, stretchable and wearable electronics has motivated the development of this kind of technique to customizable healthcare devices, such as implantable bionic ears (Figure 5) [80]. The printed bionic ears can intricately merge biological and nanoelectronic functionalities with enhanced auditory sensing for radio frequency reception and stereo audio music reproduction [81,82,83].

The advantages and disadvantages of the commonly used 3D printing technologies for bioartificial organ 3D printing are summarized in Table 1 [84,85,86,87,88,89,90,91,92,93,94,95,96,97,98,99,100].

## 3. Synthetic Polymers for 3D Bioprinting

### 3.1. Properties of Synthetic Polymers

Synthetic polymers are human-made polymers produced by chemical reactions with adjustable chemical structures and physical properties. Most synthetic polymers have supermechanical properties unlike natural polymers. Synthetic polymers are comparatively bio-inert and cannot readily incorporate bioactive ingredients, such as cells and growth factors, directly for 3D printing because the printing processes often involve the use of organic solvents, heat and poisonous activators that may reduce the bioactivities of cells and growth factors. Some synthetic polymers are biodegradable. These polymers can be degraded by microorganisms or biological fluid in vivo [101]. The degradation rates can be tailored to suit particular biomedical applications. The commonly used biodegradable synthetic polymers include PLA, PGA, PU, PLGA and PCL, which have taken a priority role in hard tissue and organ 3D printing due to their strong mechanical properties [102]. 

The mechanical properties of synthetic polymers include tensile strength, elastic modulus, fracture toughness, fatigue and elongation percentage. With proper mechanical properties synthetic polymers can withstand internal and external tensions during 3D printing processes and in vivo implantation stages. Thus, most synthetic polymers possess many intrinsic advantages in bioartificial organ 3D printing areas compared with natural polymers. The obvious advantages include convenient synthesis, being rich in resource, easy processing, stress tolerance, light weight and low cost.

For some of the biodegradable synthetic polymers, such as PLGA and PU, their sol-gel transition temperatures are much lower than those of the natural polymers, such as gelatin, alginate and agar [61,62,63]. Temperatures of minus 20–30 °C are necessary for these synthetic polymeric solutions to be solidified and printed in layers. In contrast, very high temperatures, such as 100–200 °C, are often necessary for the bulk synthetic polymers to melt [103]. Due to the limitations of 3D printing technologies, few of the biodegradable synthetic polymers can meet all the demands for bioartificial organ 3D printing with superior structural supports, non-toxic degradation products, strong mechanical strengths and controllable degradation rates. Simultaneously printing both natural and synthetic polymers with cells encapsulated in the natural polymer hydrogels for the construction of large hierarchical structures, such as hierarchical vascular, neural and lymphatic networks, has become an inexorable trend [104,105,106,107].

### 3.2. Polycaprolactone (PCL) 

**Figure 6:**
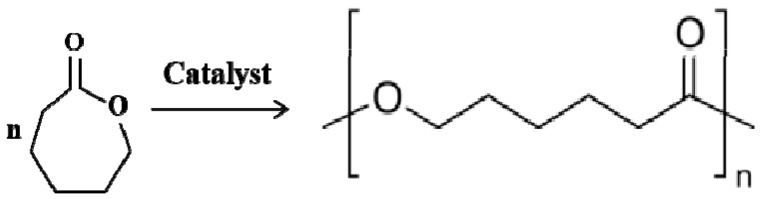
Synthesis of polycaprolactone (PCL): PCL is prepared by ring-opening polymerization of ε-caprolactone using a catalyst such as stannous octoate.

PCL is a thermoplastic polymer that has been approved by the Food and Drug Administration of USA (FDA) for use in drug delivery devices, suture materials and adhesion barriers in the human body (Figure 6) [108]. The melting point of PCL is around 60 °C, while the glass transition temperature (T_g_) of PCL is about −60 °C [93]. It is a semicrystalline and biodegradable polyester that degrades by hydrolysis of its ester linkages in physiological conditions. Compared with most natural polymers, such as gelatin, fibrin and collagen, the biodegradation rate of PCL is much slower [99].

Traditionally, PCL is frequently used as an additive for various biomedical purposes. For example, PCL is mixed with starch to lower its cost and increase its biodegradability, as an additive for resins to improve their processing characteristics and end-use properties (e.g., impact resistance), or as a polymeric plasticizer to thermoplastic PVC. The most common usage of PCL is in the manufacture of special biodegradable polyurethanes (PUs), which have been widely used as implantable biomaterials in our own group with wonderful biocompatibilities and mechanical properties [2,4,6]. During the syntheses of PUs, PCLs impart good water, oil, solvent and chlorine resistance to the produced PUs. PCL can mix with carbon black to make a printable conductive filament called carbamorph, which is useful in 3D printing of electronic sensors with inexpensive conductive materials [109].

Nowadays, PCL is regarded as an ideal structural material for FDM technologies. During the printing processes, PCL molecules maintain crystal states with low or moderate mechanical properties [110,111]. For example, Hutmacher et al. demonstrated that the mechanical properties of 3D-printed PCL scaffolds could be tailored by changing the porosities and geometries of the structures to match those of surrounding host tissues, especially in load-bearing parts, such as joint, cartilage and trabecular bone [112]. The tailored porosities and geometries of the 3D-printed PCL scaffolds were beneficial for human fibroblast proliferation, human adipose-derived stem cell (hASC) accommodation and peripheral nerve regeneration [113,114].

As most other synthetic polymers, PCL lacks natural peptide motifs that provide specific binding sites for cells. The combination of PCL with functional biomaterials or naturally derived polymers to create hybrid structures is a normal approach for PCL to be used widely. For instance, Cho et al. developed a PCL-alginate scaffold encapsulating chondrocytes and growth factors for cartilage regeneration [115]. Malda et al. fabricated a methacrylate functionalized PCL scaffold to support the cell-laden GelMA through photopolymerization [116]. After combination, the shape fidelity and mechanical strength of the printed construct, as well as the cartilage-specific matrix deposition had been enhanced. Interestingly, GelMA is a semi-synthetic polymer which consists of gelatin modified methacrylate and methacrylamide groups. The methacrylate and methacrylamide groups can be quickly photo-crosslinked under the action of blue or ultraviolet light with the existence of initiators during the 3D printing process, providing shape fidelity and structural stability at physiological temperature. As a result, the higher the GelMA concentration is, the higher the hardness of the 3D-printed objects. The gelatin motifs in the hydrogel can provide cells with an amicable biological environment for survival. Other attempts include simultaneously printing cell-laden hyaluronic acid, collagen or fibrinogen solutions in PCL scaffolds to improve structural stabilities and cell behaviors [117].

### 3.3. Polyurethane (PU)

**Figure 7:**
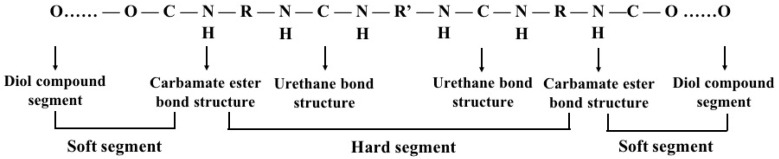
The structure of polyurethane (the urethane groups –NH–(C=O)–O– link the molecular units for polyurethane synthesis).

PU is a group of linearly segmented polymers which are composed of oligodiol (i.e., soft segment) and organic (i.e., hard segment) units through carbamate (i.e., urethane) links (–NH–(C=O)–O–) (Figure 7). PUs can be biodegradable or non-biodegradable which have been widely used in biomedical applications due to their supper mechanical properties and excellent biocompatibilities [118]. The physicochemical and physiochemical properties of PUs, such as thermosensitivity, PH-sensitivity and biodegradability, depend on their chemical compositions [119]. For example, waterborne PU is often synthesized by introducing ionic hydrophilic groups that transform PU to an ionomer and make it disperse in water. The thermosensitivity of PU hydrogels is strongly dependent on the soft segment oligodiol compositions [120]. Acrylate groups serve as an ultraviolet (UV) curing site, and can be incorporated into thermosensitive PUs for cell and tissue 3D printing.

Most of the traditionally used PUs are bioinert and non-biodegradable, and have limited applications in organ 3D printing areas. Recently, biodegradable PUs have been extensively exploited by our own group and others for bioartificial organ 3D printing due to their excellent mechanical properties, tunable chemical structures and super biocompatibilities. For example, an FDM technique was used to print the abovementioned waterborne thermosensitive PU (i.e., 80% PCL + 20% poly (D, L-lactide, PDLLA) at 37 °C for center nerve repair [121]. Neural stem cells (NSCs) (cell density 4 × 10^6^ cells/ml) could be embedded within the waterborne PU hydrogel with appropriate stiffness and showed comparable viability and differentiation capacity after 3D printed [122]. Neural-like constructs fabricated from human fibroblasts co-printed with FoxD3 plasmids in the PU hydrogel could be applied for neuro regeneration. Waterborne thermoresponsive PU hydrogel synthesized by Ho et al. could be used as a ‘bioink’ to directly reprogram cells for customized cartilage tissue engineering [123]. 

In our previous studies, a brand new biodegradable elastomeric PU was developed and 3D printed subsequently as supportive templates for cell accommodation, growth, immigration and proliferation [13,21,124]. Over the past 10 years, this biodegradable elastomeric PU has been applied in a wide range of biomedical applications, such as peripheral nerve repair conduits, rabbit vein restoration overcoats and hierarchical vascular/nerve networks [13,21,60,61,62,63,125,126]. In particular, a double-layer PU-collagen conduit was 3D printed as bridges and guidances between the proximal and distal stumps for large peripheral nerve damage repair with excellent hydrophilicity, biocompatibilities and mechanical properties [127,128].

Notably, a tubular PU-adipose stem cells (ASCs)/gelatin/alginate/fibrinogen construct was built using a double-nozzle, low-temperature deposition 3D printer at −20 °C [129]. The ASCs survived the freeze 3D printing stage by incorporating cryoprotectants, such as dextran-40, glycerol and dimethyl sulfoxide (DMSO), in the hydrogels. Cell activities were effectively preserved below −80 °C for more than 1 month. After thawing, cell viability in the hydrogels with 5% DMSO was nearly 80%, which was significantly higher than other cell preservation techniques. The cryoprotectants could reduce the risk of damage to cells through avoiding ice crystal formation during the freezing and thawing processes. The cryoprotectant incorporating 3D printing technology consequently becomes a simple, easy, labor-saving and useful method for bioartificial organ preservation in the future [62]. 

Furthermore, a hybrid hierarchical PU-cell/hydrogel construct was automatically created using our home-made double-nozzle low-temperature deposition 3D printer [21,130,131]. The incorporated biodegradable elastomeric PU, consisting of PCL and poly (ethylene glycol) (PEG), had excellent biocompatibility and tunable biodegradation property. A bioreactor was applied for pulsatile cultures of the 3D vascular templates with a principle axis. Further studies have been carried out for vascular and neural network building simultaneously in a complex bioartificial organ.

### 3.4. Polyethylene Glycol (PEG)

PEG, also known as poly(oxyethylene) or poly (ethylene oxide) (PEO), is a hydrophilic, biocompatible, non-immunogenic synthetic polyether with a linear and branched structure that has been approved in biomedicine by the FDA as a good candidate for cell encapsulation. PEG hydrogels are naturally nonbiodegradable, but can be altered to enhance degradation by incorporating degradable segments. PLA, PGA and PCL are the most commonly used hydrolytical blocks [132,133]. 

PEG and its derivatives are probably the most explored synthetic polymer for soft tissue repair. PEG hydrogels alone cannot provide an ideal environment to support cell adhesion and tissue formation due to the lack of cell-adhesive domains. Nevertheless, two hydroxyl groups of PEG-diol in the PEG molecules can be tailored into other functional groups (i.e., acrylate, thiol, carboxyl) by physical, ionic or covalent crosslinking, which are versatile for hydrogel formation or for conjugating with biomolecules [134]. PEG is often chemically modified with acrylate groups to create the photopolymerizable PEGDA in which cells can quickly be encapsulated [135]. PEGDA photopolymerized by UV-light have been applied in extrusion-based 3D printing areas for creating tissue engineering scaffolds with increased mechanical properties.

**Figure 8:**
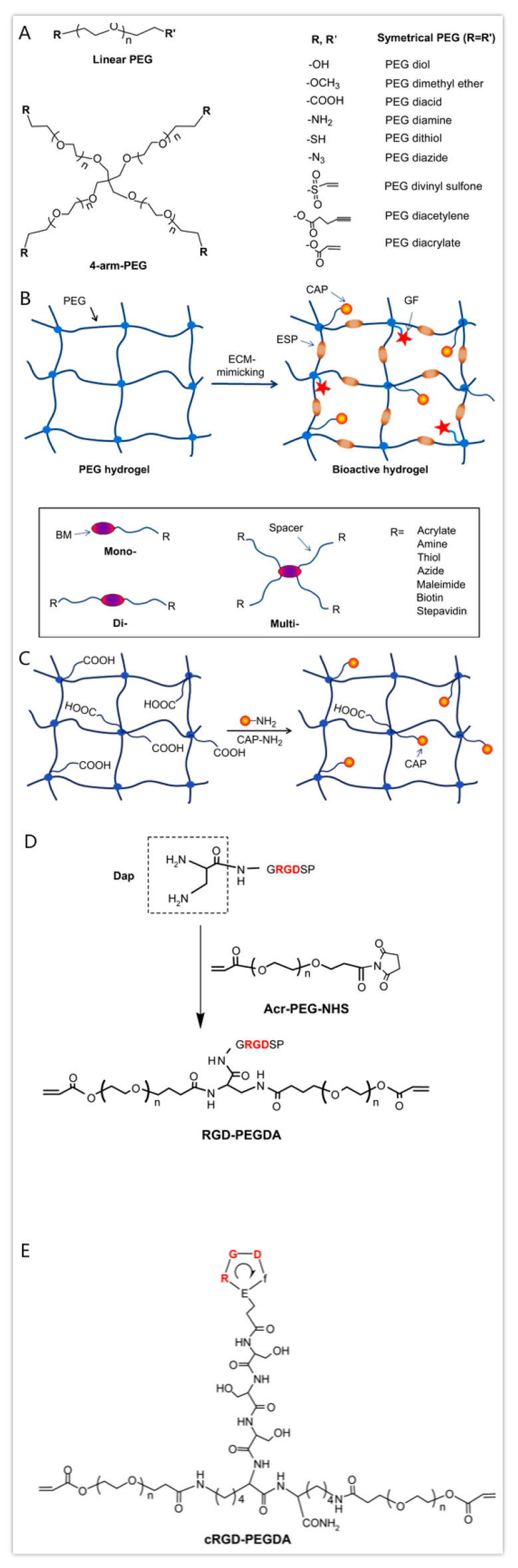
Structure and modification of polyethylene glycols (PEGs) [136]. (**A**) Structures of linear PEG and 4-arm-PEG with various functional end groups. (**B**) Bioactive modification of PEG hydrogels with bioactive molecules (BMs), such as cell-adhesive peptide (CAP), enzyme-sensitive peptide (ESP) and growth factor (GF), and major types of bioactive monomers from mono-, di- and multi-functionalization of BMs with various groups. (**C**) Fabrication of cell-adhesive PEG hydrogels by copolymerization of PEGDA and acrylic acid, followed by post-grafting of cell-adhesive peptides (CAPs) on the hydrogel surface through the reaction between the N-terminal amino groups of CAPs and the carboxyl groups provided by acrylic acid from the hydrogel. (**D**) Synthesis of RGD-containing PEGDA (RGD-PEGDA). (**E**) structure of cRGD-containing PEGDA (cRGD-PEGDA).

Recently, bioactive modification of PEG hydrogels has emerged as an important strategy to modulate specific cellular responses. Biomolecules including cell-adhesive peptides (CAPs), enzyme-sensitive peptides (ESPs) and growth factors have been employed to provide cells with an ECM-mimetic environment (Figure 8) [136]. The bioactive PEG hydrogels and their preparation approaches were reviewed by Zhu in 2010. Villanueva et al. investigated the role of cell-matrix interactions by dynamic mechanical loading in cartilage bioprinting using Arg-Gly-Asp (RGD)-incorporated PEG hydrogels. Within the mechanical stimulation, the RGD-incorporated PEG hydrogels could enhance the chondrocyte phenotype and matrix synthesis, indicating that cell-matrix interactions mediate cell activities through 3D printing [137,138]. Some researchers have combined PEG with GelMA to generate optimal hybrid ‘bioinks’ to improve the mechanical properties of the engineered hard tissues. Significant promotion of mesenchymal stem cell differentiation into cartilage and bone was observed using inkjet-based printing technologies, together with CAPs, ESPs and growth factors [139,140].

### 3.5. Polylactic-co-glycolic Acid (PLGA)

**Figure 9:**
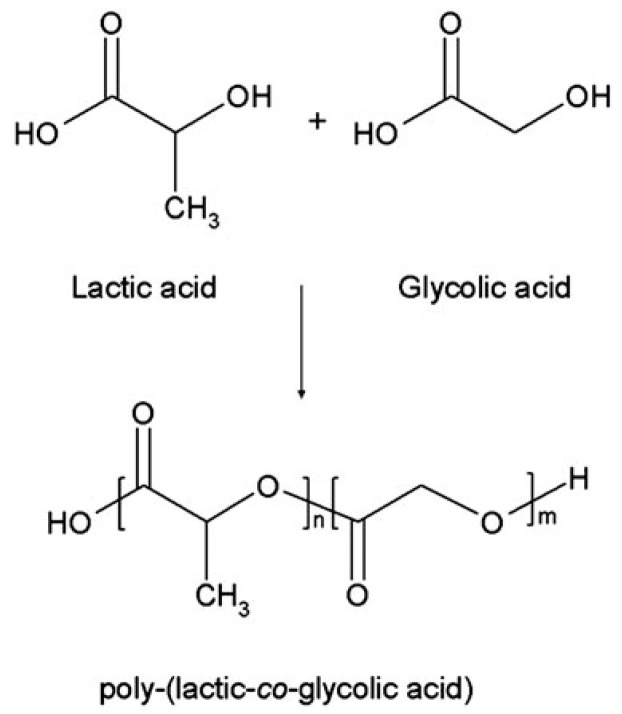
Chemical structures of poly (lactic-co-glycolic acid) and its monomers [141].

PLGA is a synthetic copolymer of lactic acid (LA) and glycolic acid (GA), which is synthesized by means of ring-opening co-polymerization of two different monomers, the cyclic dimers (1,4-dioxane-2,5-diones) of glycolic acid and lactic acid (Figure 9) [141]. Common catalysts used in the preparation of PLGA include tin (II) 2-ethylhexanoate, tin (II) alkoxides, and aluminum isopropoxide. During polymerization, successive monomeric units of lactic acid or glycolic acid are linked together by ester linkages [142]. In contrast, PLGA degrades by hydrolysis of its ester linkages in the presence of water. It has been shown that the time required for degradation of PLGA is related to the monomers’ ratio used in the starting materials (i.e., reactants): the higher content of glycolide units, the shorter the time required for degradation compared to predominantly lactides [143,144]. The final degradation products of PLGA are acidic materials, including lactic acid and glycolic acid, or innocuous salts, including lactate (salt form of lactic acid) and glycolate (salt form of glycolic acid). 

PLGA has been widely used as films, porous scaffolds, hydrogels, or microspheres in biomedical applications, approved by the FDA of the United States. PLGAs typically show a glass transition temperature in the range of 40–60 °C. It is reported that the Tg of PLGA decreases with a decrease of LA content in the copolymer and molecular weight [20,145]. Generally, PLGA 75:25 is the most widely used copolymer, consisting of 75% lactic acid and 25% glycolic acid. Compared with natural polymers, PLGA has favorable mechanical properties for load-bearing applications. Due to the poor bioactivities (e.g., osteoconductive and osteoinductive capabilities), most of the PLGAs have been printed as supportive structures with tailored mechanical strengths to provide cell-laden natural polymeric hydrogels with suitable cell growth environments. 

In 2010, a PLGA-sandwiched cell/fibrin tubular construct was fabricated in our group using a step-by-step mold/extraction method [19,146]. The inner and outer layers were made of PLGA supporting structures with different pore sizes, which played an essential role in the long-term structural stabilization and prevented excessive expansion during mechanical stimulations, while the middle layer was a fibrin-encapsulated cell hydrogel, providing an accommodation for cells to proliferate, migrate and differentiate inside. A pulse bioreactor with an adjustable 0–0.2 MPa pressure, 0–7% pulse amplitude, and a 0–80 times/min pulse frequency was developed to mimic the liquid movement in the natural blood vessels [147]. It was found that the three-layer PLGA-sandwiched structure could withstand a maximum axial stress of 1100 kPa that is significantly higher than human blood pressure in both contracting and stretching stages. Otherwise, it is hard for the cell-laden fibrin hydrogel to withstand any extra mechanical stimulation. ASCs in the middle fibrin hydrogel were induced into smooth muscle cells and arranged regularly under the growth factor inducement and dynamic training conditions. This strategy holds the promise to be widely used in complex organ manufacturing areas.

Additionally, a low-temperature 3D printing technique was also created in our group employing PLGA and other synthetic polymers to build complex hybrid 3D constructs [148]. The complex hybrid constructs are strong enough (i.e., holding sufficient mechanical properties) to support the cell-loading fibrin-based hydrogels inside the predefined synthetic polymer channels. A step-by-step cocktail induction procedure was designed to engage or steer the ASCs in the synthetic polymer channels towards both endothelial and smooth muscle cell lineages. ASCs encapsulated in the fibrin-based hydrogels were consecutively differentiated to endothelial and smooth muscle cell lineages, successfully, corresponding to their respective locations in the construct mimicking those vascular structures in a native organ.

### 3.6. Pluronic Acid (or Poloxamer)

**Figure 10:**
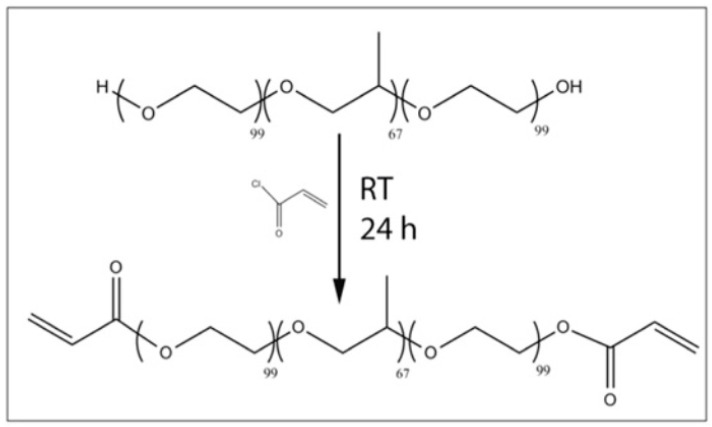
Chemical structure of Pluronic F127 before and after reaction with acryloyl chloride [149].

Pluronic acid (trade name: Pluronic^®^) is a tri-block copolymer consisting of a hydrophobic poly (propylene oxide) (PPO) segment and two hydrophilic poly (ethylene oxide) (PEO) segments arranged in a PEO-PPO-PEO configuration. This is a new class of high molecular non-ionic surfactants, with a general formula: HO(CH_4_O)_a_(C_3_H_6_O)_b_(C_2_H_4_O)_c_H, a synthetic thermoplastic polymer, in which the gelation temperature is closely related to its concentration and structure (i.e., the ratio of PPO/PEO, the PPO/PEO block lengths and the total polymer chain length) (Figure 10) [149].

Due to the thermal response property, pluronic acid has become a scaffold material during the earlier extrusion-based 3D printing development stage [150]. The low viscosity of pluronic acid at low temperatures allows it to be homogeneously mixed with cells and other biopolymers and printed as ‘bioinks’ at liquid state with special shear thinning behaviors.

The advantage of using pluronic acid as ‘bioinks’ for 3D printing is the high resolution of the printed construct. Nevertheless, the weak mechanical properties, quick degradation rates, rapid dissolution in aqueous solutions, and poor cell viabilities have greatly limited its application in bioartificial organ 3D printing. In order to alter its mechanical properties and degradation rates, acryloyl is often incorporated into the terminal hydroxyl moieties of pluronic acid through UV photo-crosslinking. 

Pluronic acid itself can also be chemically modified with other polymers to improve the structural and mechanical properties. It is reported that cell survival capability in the pluronic acid hydrogel is poor. When pluronic acid was employed for the printing of mesenchymal stem cells, the cell survival rate was very low. More than 90% of cells died on the third day when bone marrow stromal cell-laden pluronic acid ‘bioinks’ were printed [151]. Strategies to improve cell viability include diacrylation of pluronic acid and incorporation of bioactive molecules into the pluronic acid. For example, Michael et al. modified pluronic acid by mixing acrylate in the ‘bioinks’ and printing via UV crosslinking [152]. The new hydrogel was able to significantly increase the cell viability which attained 86% on day 14. Similarly, Müller et al. combined photo-crosslinkable PEG-fibrinogen with unmodified pluronic acid to create a nanostructured biosynthetic hydrogel scaffold, which could control cellular morphogenesis and behaviors to some degree [153,154].

### 3.7. Polydimethylsiloxane (PDMS)

**Figure 11:**
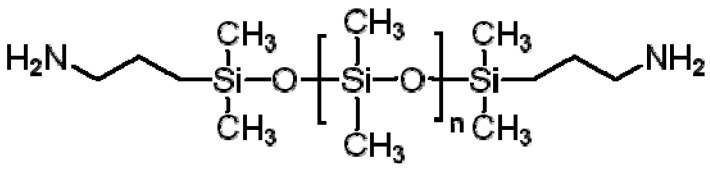
Structural formula of polydimethylsiloxane.

**Figure 12:**
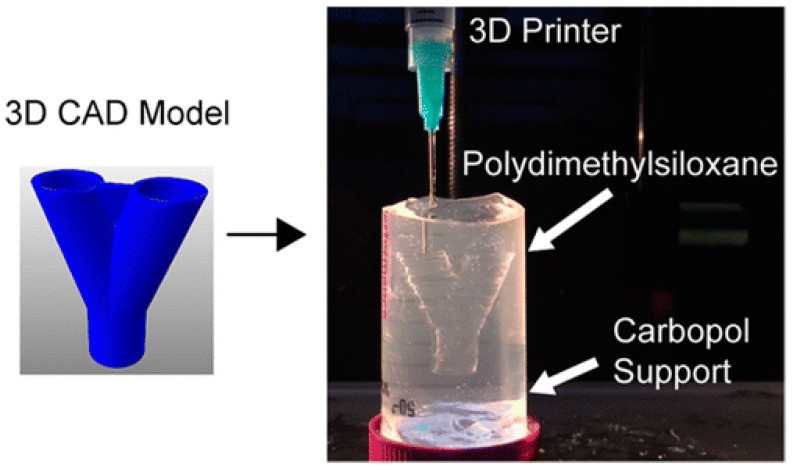
Three-dimensional printing of hydrophobic polydimethylsiloxane prepolymer resins within a hydrophilic Carbopol gel support via freeform reversible embedding [156].

PDMS, also called silicone, is a biocompatible transparent rubber or elastome (Figure 11) [155]. With the recent advances in 3D printing, PDMS can be used for complex shape building. It can be stereolithographically printed into microfluidics, cell culture substrates and flexible electronics. In one of the current studies, PDMS was printed in a Carbopol. The Carbopol acts as a Bingham plastic that yields and fluidizes when the syringe tip of the 3D printer moves through it and solidifies the PDMS extruded within it (Figure 12) [156]. The immiscibility of hydrophobic PDMS in the hydrophilic Carbopol confines the PDMS prepolymer within the support for curing times of up to 72 h while maintaining dimensional stability. After printing and curing, the embedded PDMS was released from the Carbopol support hydrogel through phosphate-buffered saline solution. By implementing low-cost open-source hardware and software tools, the printing technique can be rapidly adapted for biomedical applications.

Unlike most other polymers which become more viscous under pressure, PDMS has the opposite, non-Newtonian response. This is a benefit for 3D printing because it is expected that a fluid is viscous enough to sit in the nozzle before being pushed out with less viscosity. As soon as the PDMS leaves the nozzle, it regains the original viscosity and the printed threads retain their shapes [157,158].

## 4. Typical Organ 3D Printing Technologies

Over the last decade, a packet of organ 3D printing technologies have been developed, being regarded as cutting-edge technologies, to construct bioartificial organs mimicking the anatomical and functional features of native organs. These cutting-edge technologies have made it possible to precisely place multiple cell types along with other biomaterials in a single 3D construct. They are so far the most attractive and powerful tools to manufacture bioartificial organs with complex geometrical and functional similarities to human organs in biomedical fields [159,160,161]. 

From the manufacturing point of view, organ 3D printing is a fast prototyping procedure that builds bioartificial organs under the instructions of predesigned CAD models with the deposition of cell-laden natural and/or synthetic polymers through a layer-by-layer methodology. Each deposited layer represents a cross section of the organs derived from the virtual CAD models and in turn is printed over the previous, so that the final product is an approximation of the predesigned CAD model, which has been referred to as organ analogue or precursor in our earlier studies [19,72]. 

### 4.1. Typical Organ 3D Printing Steps 

Similar to building a ‘nuclear plant’, a typical organ printing process encompasses three major steps: (1) starting material preparation and organ architectural (i.e., blueprint) predesigning; (2) 3D printing using cell-laden natural hydrogels and supportive synthetic polymers, such as heterogeneous cells/extracellular matrices (ECMs) and stem cells, ECM-like polymers or growth factors; (3) maturation of homogeneous and heterogeneous tissues in the 3D printed construct, a post multicellular organization, homogeneous/heterogeneous tissue modulation, coordination and maturation for the expected physiological or pathological functionality realizations [2,4,5]. All the major three steps depend largely on the processing material selection and 3D printing technology utilization. 

In the first step, the selection of biocompatible cell types and implantable polymers are critically important, which is closely related to the vice reactions, such as infection, fibrous encapsulation, toxic degradation product after implantation. It also involves collecting information about the defective or failed organs, translating those data into mathematical CAD models, and subsequently feeding those data into the available 3D printers. Or using CAD models for overall organ architectural (i.e., blueprint) predesigning. Some scientists, such as Vladimir Mironov, Ali Khademhosseini and Jennifer Lewis, have paid attention to this step through different 3D printing technologies, such as inkjet printing, extrusion printing and laser- or light-assisted printing [8,102,160]. Other pertinent technologies, such as FDM [14], selective laser sintering [15], digital light projection [16], thermal fusion bonding [17], high temperature sintering [18] and photo-crosslinking [19], have also been developed quickly for nearly every human organ, such as kidney, liver, lung and brain, construction respectively. 

It is realized that the selected polymers should be biocompatible, biostable, bioprintable. Most of the naturally derived polymers, such as gelatin, alginate, hyaluronan, fibrinogen, agarose, can dissolve in water-based liquid (i.e., cell-friendly inorganic solvents) and form solutions. These solutions can entrap cells, growth factors and drug molecules in mild conditions. However, few of the cell-laden natural polymers can be printed in layers and physically translated into hydrogels with mechanically stable constructs. This is because that except gelatin and agarose, nearly all the natural polymers need to be crosslinked to maintain structural integrity. The crosslinking processes can be chemical, enzymatic or photic (including ultraviolet, UV). Meanwhile, the phase transition (i.e., sol-gel transformation) temperatures for most of the natural polymer solutions are too low to form solid structures at facile or room temperature. The cell-laden natural polymers are hard to print in layers using normal 3D printers under cell endurable temperatures [61,62,63]. Synthetic polymers are often needed to support or protect the cell-laden natural polymer constructs. Some of the synthetic polymers need to be deposited through low-temperature manufacturing systems via additional nozzle modeling and cell cryopreservation technologies [2,4,6]. 

In the second step, 3D printing is done under the instruction of CAD models with a suitable 3D printer. Heterogeneous adult cells/extracellular matrices (ECMs), stem cells, ECM-like polymers and growth factors are printed together to form multiple tissue contained organs. Beside cell-laden natural or synthetic polymers, cellular spheroids and nanofibers can also be printed in a freeform fashion. At present, a few 3D printing technologies have been exploited to fulfill different assignments for bioartificial organ manufacturing with a variety of specific physiological functions [61,62,63].

In the third step, multicellular organize, homogeneous/heterogeneous tissue modulate/coordinate/maturate for the expected physiological or pathological functionality realization. The bioartificial organs are temporarily cultured in vitro or in vivo after 3D printing. For in vitro pulsatile cultures, sophisticated bioreactors allow the vascular networks to be fully endothelialized [2,4,6]. Synthetic polymer incorporation can make the living constructs anti-suturable and perfusable, so they can be connected directly to the host blood vessels to restore the missing physiological functions. This strategy has provided a basic mechanism for producing implantable bioartificial organs, which is hard to achieve using traditional tissue engineering, biomaterial and cell transplantation approaches. It is so far the easiest and safest way for bioartificial organs manufacturing.

### 4.2. Double-Nozzle Low-Temperature Organ 3D Printing 

Theoretically, any polymers which have a sol-gel phase transition character can be applied in 3D printing fields. In fact, very few polymers can be printed in layers at room temperatures. Most of the sol-gel transition temperatures of synthetic polymers are much lower than those of natural polymers. For example, the sol-gel transition temperatures for PLGA and PU are minus 20–30 °C. In another word, temperatures of minus 20–30 °C are necessary for these synthetic polymeric solutions to solidify. However, cell metabolism activities are interrupted or even stopped under these low temperatures. The combination of cell cryopreservation and 3D printing technologies is a key step towards safeguarding living cells under low temperatures [15,16].

**Figure 13:**
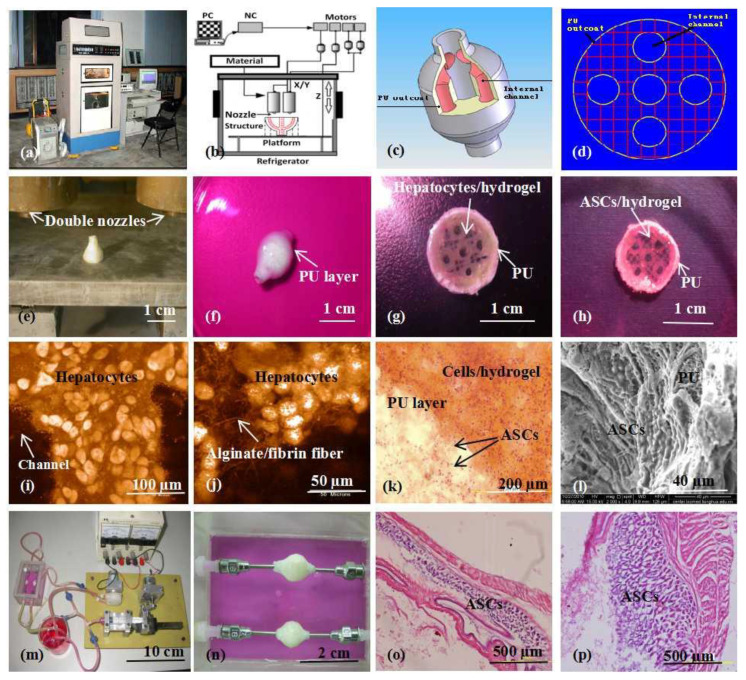
A large scale-up 3D printed complex organ with vascularized liver tissue constructed through the double-nozzle 3D printer created in Tsinghua University, Prof. Wang’s laboratory: (**a**) the double-nozzle 3D printer; (**b**) a computer-aided design (CAD) model containing a branched vascular network; (**c**) a CAD model containing the branched vascular network; (**d**) a cross section of the CAD model containing five sub-branched channels; (**e**) working platform of the 3D bioprinter containing two nozzles; (**f**) an ellipse sample containing both a cell-laden natural hydrogel and a synthetic polyurethane (PU) overcoat; (**g**) several layers of the ellipse sample in the middle section containing a hepatocyte-laden gelatin-based hydrogel and a PU overcoat; (**h**) several layers of the ellipse sample in the middle section containing an adipose-derived stem cell (ASC)-laden gelatin-based hydrogel and a PU overcoat; (**i**) hepatocytes encapsulated in the gelatin-based hydrogel; (**j**) a magnified photo of (**i**), showing the alginate/fibrin fibers around the hepatocytes; (**k**) ASCs encapsulated in the gelatin-based hydrogel growing into the micropores of the PU layer; (**l**) ASCs on the inner surface of the branched channels; (**m**) pulsatile culture of two ellipse samples; (**n**) two samples cultured in the bioreactor; (**o**) static culture of the ASCs encapsulated in the gelatin-based hydrogel; (**p**) pulsatile culture of the ASCs encapsulated in the gelatin-based hydrogel. Images reproduced with permission from [162].

For bioartificial organ manufacturing, a series of pioneering studies have been done in our group using our home-made double-nozzle low-temperature deposition manufacturing (DLDM) 3D printer (Figure 13) [162]. Before 3D printing, cryoprotectants, such as DMSO, glycerol and dextran-40, need to be incorporated into the gelatin-based hydrogels for cell survival through the freezing and thawing procedures [63]. Both cell-encapsulating natural polymer hydrogels and synthetic polymer solutions have been designed to undergo a rapid liquid-to-solid phase transition in the presence of living cells. Two distinctive material systems, including natural polymers dissolved in inorganic solvents and synthetic polymers dissolved in organic solvents, have been printed simultaneously into predefined space to form complex 3D architectures under the instruction of CAD programs at low temperatures (i.e., minus 20–30 °C). Compared with the former pure cell-laden natural polymer 3D printing technologies, the DLDM system represents a major advance and a significant step forward in large scale-up vascularized organ manufacturing which has perplexed tissue engineers for more than three decades [2,4,6].

Importantly, utilizing the advantages of this DLDM system, synthetic polymers, such as the PLGA, PU with excellent mechanical properties, and natural gelatin-based hydrogels, such as the gelatin/alginate, gelatin/fibrinogen, gelatin/alginate/fibrinogen with super cell compatibilities, have been successfully printed together, creating scale-up hierarchical vascular templates [61,62,63]. This means that all the following bottleneck problems encountered by tissue engineers and other scientists for more than several decades have been solved using this unique DLDM system.

Firstly, the novel DLDM system has significantly enhanced the processable biomaterial scopes [163]. Both synthetic and natural polymers and their derivatives can be printed simultaneously and precisely under the predefined CAD models. Different material systems with inorganic and organic solvents can fuse and connect tightly through adjusting the printing parameters. A wide range of biomaterials, such as cells, growth factors, drugs, bioactive agents, little molecular chemicals (e.g., tricalcium phosphate and hydroxyapatite), can be incorporated into the organic and inorganic polymer systems directly for different tissue and organ (e.g., hard tissue and organ) manufacturing.

Secondly, the novel DLDM system has significantly enhanced the mechanical properties, shape fidelities and structural integrities of the 3D printed constructs [163]. With the support of the synthetic polymers, the cell-laden natural polymer systems and hybrid cell-laden natural/synthetic polymer systems are much more stable in the predefined 3D constructs. Remarkably the stability of the gelatin-based hydrogels has been obviously prolonged when the cell-laden constructs are cultured in a liquid medium at 37 °C. Evidently, the 3D environments created allow the cells to thrive over longer periods for both in vitro culture and in vivo implantation before the synthetic polymers are biodegraded. Typically, most of the PLGAs and PUs we have used are biodegraded in about half a year. 

Thirdly, the novel DLDM system has significantly enhanced the tissue/organ generation (i.e., formation) and application (e.g., implantation) rates [163]. With the existence of synthetic polymers, the concentrations of the gelatin-based hydrogels can be substantively reduced, meanwhile the cell density in the gelatin-based hydrogels can be drastically augmented. Most importantly, the 3D constructs in which cell-laden natural hydrogels wrapped in the synthetic polymers can be anastomosed to the host vasculature with the prominent anti-suture properties. This can distinctively ameliorate the cell survival environments and sagaciously aggrandize the defective/failed organ restoration ratios. It is therefore being regarded as a long-awaited breakthrough in tissue engineering, biomaterials, cell transplantation and other pertinent biomedical fields [143,144,145,146,147,148]. 

### 4.3. Combined Multi-Nozzle Organ 3D Printing Technologies

**Figure 14:**
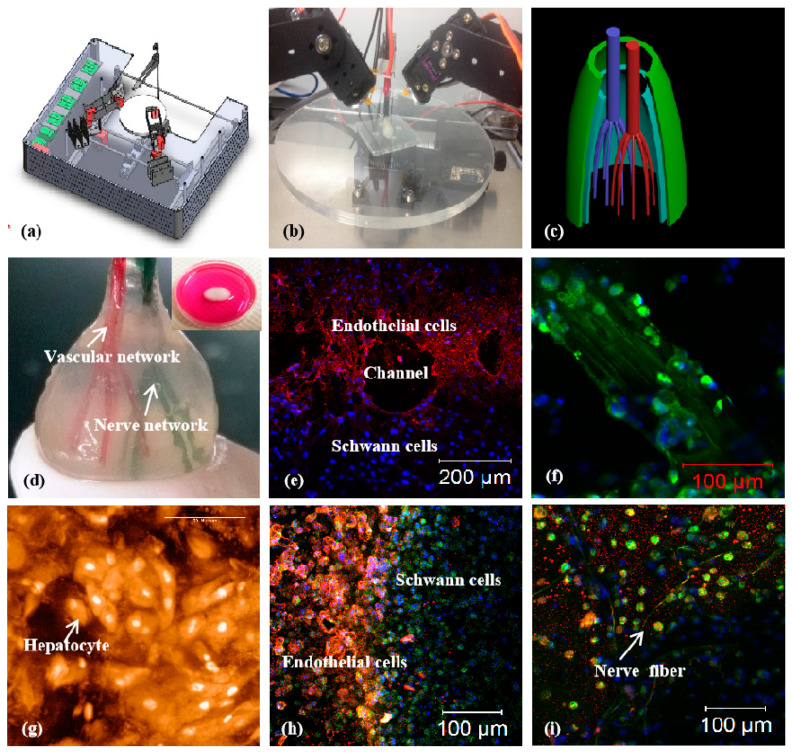
A combined four-nozzle organ three-dimensional (3D) printing technology created in Tsinghua University, Prof. Wang’ laboratory in 2013: (**a**) equipment of the combined four-nozzle organ 3D printer; (**b**) working state of the combined four-nozzle organ 3D printer; (**c**) a computer-aided design (CAD) model representing a large scale-up vascularized and innervated hepatic tissue; (**d**) a semi-ellipse 3D construct containing a poly (lactic acid-co-glycolic acid) (PLGA) overcoat, a hepatic tissue (made from hepatocytes in the gelatin/chitosan hydrogel), a branched vascular network (with a fully confluent endothelialized adipose-derived stem cells (ASCs) on the inner surface of the gelatin/alginate/fibrin hydrogel) and a hierarchical nervous (or innervated) network (made from Schwann cells in the gelatin/hyaluronate hydrogel), the maximal diameter of the semi-ellipse can be adjusted from 1 mm to 2 cm according to the CAD model; (**e**) a cross section of (**d**), showing the endothelialized ASCs and Schwann cells around a branched channel; (**f**) a large bundle of nerve fibers formed in (**d**); (**g**) hepatocytes underneath the PLGA overcoat; (**h**) an interface between the endothelialized ASCs and Schwann cells in (**d**); (**i**) some thin nerve fibers [164].

In 2013, a combined four-nozzle extrusion-based 3D printer was explored in our laboratory for more complex organ manufacturing (Figure 14) [164]. Obviously, this 3D printer is equipped with more upgraded software and hardware, which can deliver four kinds of biomaterials, including supportive materials, at least three heterogeneous living cells and growth factors, simultaneously to formed 3D constructs with high structural fidelity and cell survival rate. Cells can survive the heterogeneous material processing, polymerization/crosslinking and storage stages with the available RP techniques. 

Compared with the abovementioned DLDM, the four-nozzle extrusion-based 3D printer holds the capability to merge more merits of different biomaterials to generate bioartificial organs with many more physiological functions and clinical requirements for implants [163,164]. The common physiological functions of a solid organ, such as containing at least two large-volume homogeneous and heterogeneous tissues, anti-suture hierarchical vascular and neural networks can be accomplished in one program. With the upgraded software and hardware, four completely different material systems have been integrated properly as an organic entity under the instruction of CAD models. ASCs, hepatocytes, Schwann cells and PLGA are first printed into complex organs via different nozzles, such as the extrusion-based and injection-based nozzles. Three homogeneous and heterogeneous tissues along with a synthetic PLGA overcoat coordinate in a predefined manner. The predefined geometrical structures, ECM-like polymer compositions, and physiological functions can be conveniently adjusted according to the CAD models as well as the processing parameters. A large number of complex bioartificial organs, such as the kidney, brain, bone, and skin, containing both vascular and neural networks can be hence constructed. 

Evidently, the number of cell types that can be simultaneously printed into an implantable 3D construct depends largely on the nozzle numbers of the 3D printer. The combined multi-nozzle 3D printer is therefore outstanding in its capabilities to offer more methodologies and facilities to control the heterogeneous cellular activities, tissue morphologies and organ functions [164]. It is capable to precisely control the internal/external architectures, such as the channel size, shape, orientation, branching and topology, material constitutes, such as the hepatocyte-laden gelatin/chitosan, ASC-laden gelatin/alginate/fibrinogen, and Schwann cell-laden gelatin/hyaluronate, growth factor concentrations in the gelatin-based hydrogels, and physiological functions, such as the anti-suture vascular and nervous networks, which cannot be realized using other available technologies [61,62,63].

It is worth noting that the combined four-nozzle extrusion-based 3D printer is hitherto the most sophisticated 3D printing technology for complex bioartificial organ manufacturing. With the development of the combined multi-nozzle organ 3D printer, more heterogeneous cell types, growth factors and gradient ECM-like polymers have been concurrently printed at the connective positions, mimicking their respective positions in a natural organ, such as the liver [164]. Superior to the earlier reported single-nozzle and DLDM RP technologies, the combined four-nozzle 3D printer can create more complex geometrical, material and functional features in a complicated 3D construct. More cell lines can be incorporated along with the unique gelatin-based hydrogels. EGF growth factors can be mixed into the ASC-laden gelatin/alginate/fibrinogen hydrogels directly for stem cell endothelization. More optimal environments for heterogeneous cell-cell, cell-ECM-like polymeric matrices and cell-growth factor interactions can be achieved. It is expected that with the additional nozzles in the 3D printer, more special characteristics of human organs, such as the nephric tubules in the kidney, the alveolus pulmonis in the lung, and the lactiferous ducts in the breast, can be realized in a predesigned 3D construct under a precisely controlled manner. The outstanding combined multi-nozzle organ 3D printer therefore holds the promise to eventually manufacture all human organs with a spectrum of many more physiological functions [165]. This is another outstanding contribution of our group to people all over the world. There will be no doubt that the advanced high technologies can significantly improve the life quality and prolong the average lifespan of human beings in the near future. 

## 5. Challenges and Perspectives

The need for organ substitutes in patients continues to increase because of a scarcity of donors, as well as biocompatibility issues in transplant immune rejection. To address these issues, scientists have automatically printed bioartificial organs as an alternative to implantation. Organ 3D printing promises to produce bioartificial organs with predesigned geometrical architectures, heterogeneous material components, and multiple physiological functions, similar to those of native human organs. To date, several extrusion-based 3D printing technologies have exhibited their unique and outstanding capabilities for both hard and soft organ manufacturing [166,167]. Nevertheless, organ 3D printing still has unsolved challenges to overcome. A few critical challenges should be addressed before using cell-laden natural and synthetic polymers for organ 3D printing. In this section, the current technical challenges and future prospects are reviewed.

First of all, the creation of a hierarchical vascular network (i.e., vasculature) mimicking those in a native human organ is very difficult because the vasculature has gradient structures, such as large arteries, small arteries, capillaries, small veins and large veins; heterogeneous tissues, such as endothelium, smooth muscle, and fibrous tissues with different properties, such as high elasticity, flexibility and anticoagulant properties. At present, most of the existing 3D printing technologies can be used to precisely dispense one or two cell-laden hydrogels for the construction of simple tissues or vascularized tissues. They are far from recapitulating the intrinsic complexity of a vasculature in a natural organ. Despite the substantial progress in this field during the past few years, building a real blood circulatory network containing stable large vessels, small vessels and capillaries inside the cell-laden hydrogels remains a critical challenge. It is especially difficult to control the highly branched arterial networks with ubiquitous geometrical, material and functional features [168,169]. 

Another critical challenge in future organ 3D printing is that it needs to provide personalized organs, with customized designs, cell types, polymer components and bioactive agents. Anatomical and physiological specifications for each individual patient will become the mainstream for organ restoration and drug screening. Several extrusion-based 3D printing technologies have been used to mimic the micro- and macroarchitectures of some solid organs, such as the liver, heart, and kidney. Successful building of hierarchical vascular, neural, lymphatic, even biliary networks in a 3D printed construct is ultimately necessary. The implantable organs can be built by 3D scanning the symmetrical ones and converting the data to an anatomically matched construct. The imaging-coupled 3D printing approaches will definitely make the customized organ manufacturing and restoration dreams come true in a fast, easy and repeatable manner [106,107,108,109].

To overcome the limitations of the existing 3D printing technologies for complex organ manufacturing, several approaches are trying to mimic native vascular tissues with specific natural and synthetic polymers. For example, by controlling the concentration and diversification of the natural and synthetic polymers, it is expected that 3D printed constructs will be able to capture the specific micro- to macroenvironments of the vasculatures with desirable physiological functions [170]. Single or multiple vascular cell lines, such as endothelial cells, smooth muscle cells and fibroblasts, have been applied directly or indirectly into the 3D-printed templates for the generation of a perfusable vascular network [171]. Nevertheless, these techniques are still incapable of manufacturing a complex bioartificial organ with a whole spectrum of physiological functions, especially with respect to embodying multiple networks, such as vascular, neural, biliary and lymphatic. Additional capabilities of the current available 3D printers should be equipped [172]. 

Recently, 3D printing has become more and more economical and practical in organ blueprint planning, medical assistance equipment production and surgical guide preparation. Particularly, organ 3D printing is a major aspect of recent innovation in the fields of biomedicine, including biomaterial implantation, organ restoration, drug screening, disease molding and cell transplantation [173,174]. Those 3D printers that are easy to set up and use have provided some useful information for the printing of each particular human organ. Better updated 3D printers with higher enabling capacities are still at the development stages. The available extrusion-based and customized organ manufacturing technologies with patient-derived cells, cell-laden biocompatible hydrogels and mechanical superior synthetic polymers all have positive impacts on following organ curing strategies, such as reducing the operation courses, increasing the recovery rates and minimizing the disease miseries [175,176,177]. It is expected that organ 3D printing will drastically make the dream of patient-made production from medical image data and implantable biomaterials to clinical applications come true in the next few years. 

## 6. Conclusions

3D printing is typically a fast prototyping method that can build objects from CAD models based on the deposition of connectable materials through a layer-by-layer methodology. Recently, 3D printing technologies have become an attractive process for bioartificial organ manufacturing for a wide range of biomedical applications. Advanced 3D printers can be used for designing and manufacturing bioartificial organs with similar geometrical structures, heterogeneous material components and physiological functions to their native counterparts. Compared with the single-nozzle extrusion-based 3D printing technologies, The development of DLDM and combined multi-nozzle 3D printing technologies can sophisticatedly convert functional biomaterials into high-performance bioartificial organs in a controllable, scalable and affordable manner, presents great research opportunities and promises for the future of bioartificial organ manufacture, implantation and therapies.

Synthetic polymers have unique, superior and/or exclusive physiochemical properties compared to natural polymers during bioartificial organ 3D printing and the following implantation stages. Proper integration of synthetic polymers into the cell-laden natural polymer 3D printing process is a successful way for bioartificial organ manufacturing with a wide range of biomaterials, including living cells, growth factors, and other bioactive agents. Due to the specific properties, synthetic polymers are able to withstand internal and external tensions during organ 3D printing with multiple cell types and hierarchical vascular/neural networks. These polymers are often employed as overcoats for the assembling of multiple cell-laden natural hydrogel and the construction of hierarchical vascular/neural networks. It is undeniable that the advanced organ 3D printing technologies represented here will reshape the healthcare landscapes for the next generation of allogenic organ transplantation and bring huge benefits to human beings in the near future.

## Figures and Tables

**Table 1:** Comparison of 3D printing technologies for bioartificial organ manufacturing.

3D Printing Technique	Working Principle	Bioinks	Cell Density	Cell Viability	Printing Speed	Resolution	Cost	Advantages	Disadvantages	Ref.
Inkjet-based 3D printing	Using thermal or acoustic force to eject very small size ‘bioink’ drops onto a substrate	Thermosensitive hydrogels (e.g., PEG) and some nature polymers (e.g., alginate collagen, fibrinogen) with viscosity of 3.5–12 mPa/s	Low (<1 × 10^6^ cells/mL)	85%	Fast (1–10,000 droplets/s)	High (≈50 μm)	Low	Low cost; high printing resolution; low viscosity; fast printing speed	Poor mechanical properties; poor cell sedimentation effects; low cell densities	[84,85,86]
Fusion deposition modeling (FDM)	Molten thermoplastic materials through one or more heated extrusion heads with a small orifice in a specific lay-down pattern	Thermoplastic materials (e.g., PCL, PLA, PVA, ABS, TPU) with viscosity of 30 mPa/s to >6 × 10^7^ mPa/s	None	None	Slow (200 μm–10 mm/s)	Low (100 μm to 1 mm	Medium	Low cost; a wide range of materials; excellent mechanical properties	Only applicable for thermoplastic materials; high temperature; cannot incorporate cells, growth factors, and other bioactive agents	[87,88]
Extrusion-based 3D printing	Biomaterials are extruded though one or more nozzles under controlled pressure in a layer-by-layer pattern	Most nature polymers and some synthetic polymers (e.g., alginate, gelatin, collagen, PEG, PLGA, PU) with viscosity of 30 mPa/s to >6 × 10^7^ mPa/s	High (>1 × 10^8^ cells/mL)	40%–100%	Medium (5–20 mm/s)	Medium (10–100 μm)	Low	High cell densities; high cell viability; various printing materials; flexible geometric shapes	Only applicable for viscous hydrogels; moderate resolution	[89,90,91,92,93]
Stereolithography (SLA)	A solid freeform, nozzle-free technology based on photosensitive polymer formulation under laser beam	Photopolymers	Medium (<1 × 10^8^ cells/mL)	90%	Fast (normally 30–45 min)	High (100 μm)	Low	High printing resolution; fast printing speed; difficult to print multiple cell types	Cytotoxicity of the laser beam and photoinitiators; additional post-curing process may be necessary to remove the unpolymerized liquid resin; poor cell deposition effects	[46,94,95]
Digital light processing (DLP)	A solid freeform, nozzle-free technology based on photosensitive polymer formulation under laser beam	Photopolymers	Medium (<1 × 10^8^ cells/mL)	90%	Higher than SLA (10–50 μm)	Higher than SLA (10–50 μm)	Low	High printing resolution; fast printing speed; difficult to print multiple cell types	Cytotoxicity of the laser beam and photoinitiators; additional post-curing process may be necessary to remove the unpolymerized liquid resin; poor cell deposition effects	[96,97]
Laser-based 3D printing	Laser pulse generates a high-pressure bubble towards the collector substrate	Nature polymers (e.g., alginate, gelatin, fibrinogen) and some synthetic polymers (e.g., PCL, PLGA) with viscosity of 1–300 mPa/s	Medium (≈1 × 10^8^ cells/mL)	90%–95%	High (10–40 μm)	High (10–40 μm)	High	High printing resolution; wide range of printable viscosity; moderate cell viability	High printing resolution; wide range of printable viscosity; moderate cell viability	[98,99,100]
